# General Items Suggested by a Review of the Minutes of the Last Meeting

**Published:** 1845-09

**Authors:** 


### ARTICLE VIII.

### *General Items suggested by a Review of the Minutes of the Last Meeting. By the* Syracuse Editor.

By comparing the transactions of the American Society with the professed objects of this association, as set forth in the preamble to the constitution, what do find to be the result? A superficial glance would, perhaps, decide, that few of the objects were gained by these meetings; but we think that although they have not been as profitable to the *members of the association* as they might have been, or as they will be, when the organization of the society is complete, and they have less call for warring against quacks and quackery; yet they have been all that circumstances could admit of, and the result has been salutary, both as regards the final prospects of the society, and the general good which this association promises to the public. It could hardly be expected that the first attempt at organizing and conducting a national society should be faultless, yet it has at least served the purpose of a standard to rally under, till a more perfect one could be reared, and it must be confessed that this work of remodeling has employed the attention and time of this society much to the exclusion of that which might have been more profitable to its members, for the time being. But it was as necessary to occupy the time in this way till the organization and structure was complete, as to finish a *ship* before it was sent out to contend with the ocean's storm, and to be an instrument of profit. At the outset, there seems to have been more regard to speed than safety; and although it was found that the American Society was making headway, she was doing so at the risk of her own safety—possessed "more sail than ballast." To remedy this, has been, to a very great extent, the business of the last two or three meetings, and it tended very greatly to retard her progress in the attainment of some of her professed objects. But, from the great ability of the committee in whose hands this work of remodeling has been placed, and from the accumulated experience of this society, in respect to such laws as are safe in practice, we have great confidence to believe that very little time, after the close of the next annual meeting, need be absorbed to place this society on that firm basis which will stand the test of time and experience. As on the laws, which will doubtless be proposed by this committee, will hang the future well-being and success of this association, we hope all who feel an interest in the perpetuity of this institution will make the necessary sacrifice to attend the next annual meeting, and assist in adopting them.

Another topic which has absorbed much of the time of these meetings, is the exposition and suppression of quackery—particularly as exemplified in the use of amalgams for filling teeth. The great unanimity of feeling upon this subject, by members situated most remotely from each other, expressed in essays, prepared by them before leaving home, must have forcibly struck all who were present at the last annual meeting. It seemed as if they had unanimously agreed to write upon the same subject, and treat it in the same way—each vieing with the other in their efforts to condemn it most strongly. Dr. E. Parmly, in his opening remarks, spoke upon this topic in his usual candid and forcible style, reprobating it not only in general terms, but more particularly as connected with the American Society. Dr. P. was followed by Dr. J. H. Foster, who, in an opening address to the society, treated the same subject with an ability and earnestness calculated to force every *living* quack to abandon his way, and have rendered the prospect of a resurrection exceedingly uncomfortable to *dead* ones.

The same sentiments were expressed by most of the other essayists, from different parts of the union. This subject occupied more than half of the whole session, and the grand result of the deliberation and action would have compensated the society most richly for a month's session. This species of quackery was entirely disposed of, so far as regards the society, and received its death blow, as regards the public, without limit.

The unanimous vote to expel from the ranks of the society all who refuse to pledge themselves not to use it, and, moreover, to condemn it; together with the efficient measures adopted to test every member, and thus "divide the house," will be hailed as a bright and glorious act in the history of this body in all future time.

The circular letter upon this subject, proposed by Dr. E. Parmly, and which has had a general circulation, will do immense good; and for which, Dr. P. deserves the most cordial thanks no less of the profession than the public.

There can be no remaining doubt that this, as a time-absorbing subject, in connection with the annual meetings, is disposed of, and that to the entire satisfaction of every *worthy* member.

That there are other species of quackery which will hereafter come up for the action of the society, we have no doubt, yet we can safely assert, that by freeing our ranks from *this queen bee*, in the quack's hive, we have accomplished very much towards the extinction of the entire swarm. To suffer any one subject to absorb so much time and attention as has this, at our annual meetings, especially to the exclusion of those more pleasant and *directly* more profitable to the members, requires some good reason. This reason we have most amply in the following considerations:

1st. It is carrying out one of the direct, and most important, objects for which this association was formed.

2d. This species of quackery may be justly regarded the embodyment of all others. No man who has so little self-respect as to use this amalgam, to any considerable extent, will refuse to stoop to any species of quackery, which can contribute to his pocket; and so far as our observation has extended, there are very few who have kept aloof from *this*, that have adopted or practised *any other* species to any alarming extent. Strike from the list of practising dentists those now habitually using mineral paste as an article to plug teeth, and we venture to say, that few, if any, would remain who could not be justly regarded good practitioners. We by no means intend to say by this, that if we were to strike out *this item* in the practice of those referred to, we should make them either honorable men or good practitioners; but as we weaken public confidence in *this* vile deception, and enlighten the public mind in respect to it, we not only blot out this particular species of quackery, but, to a corresponding extent, weaken the power of these charlatans to practice this, or any other deceit, by pointing to them as the men who have, in at least one way, imposed upon the community, and filled their teeth with no higher motive than filling their own pockets, and this regardless of consequences.

3d. This kind of quackery presents to the uninstructed and unsuspecting more prima facie evidence of being worthy of confidence than any other equally dangerous.

The public, we may say, generally judge by *visible* results. If a man professing to be qualified to plug teeth, and uses *any kind of foil* for the purpose, any lack of ability will soon be discovered, by its sudden disappearance; this the public can see and appreciate, and hence such men very soon lose their ability farther to impose upon the community. Far fewer are able to judge of the healthy state of the mouth. When, therefore, a man claims that *he* has an article with which he can fill teeth, and "which *will not come out*," and "which by its great hardness, actually restores the teeth to soundness;" and when, after the lapse, perhaps, of a year, these patients find sure enough it *has not* come out, they are perfectly satisfied that mineral paste is just the thing. These people have not discovered the fact, that in each of these teeth the process of decay has been and is still going on, and that their mouths are filled with a poisonous oxyd, and, moreover, that the whole mouth is so disordered as to be past remedy.

If one professes to extract teeth without pain by cutting the dental ligament, and a patient finds that his dentist has not only broken his tooth, but perhaps the jaw, this delusion is soon over.

If a dentist professes to kill nerves "without giving pain," and treats an inflamed nerve to a dose of kreosote and arsenic, and thus confines his patient to his room a week, suffering the most excruciating agony, and he finally comes out with a discolored and ulcerated tooth, *this* kind of deception, if not the malady, has found a remedy.

A very similar remark applies to most of the malpractices now in vogue.

To say the least, most quack *operations* will sooner show the fault of the operator than the miserable and detestable practice of filling teeth with amalgam. This is, hence, the most insidious, and requires a correspondingly greater effort to suppress it. In conclusion, we feel that the American Society would have been inexcusable, had they done less to accomplish this end; nor do we believe they will be called upon to do more. Certainly no more than to carry out the action already taken upon the subject. When the attention and time of the society can be less absorbed by these subjects, more may be devoted to the discussion of subjects of practical interest; and from the many that were presented during the last meeting, in the short time devoted to them, we have very much to expect, when a sufficient time is devoted to this field of investigation.

By a special resolution, one entire day is, during the next meeting, to be devoted to the discussion of practical subjects.

In this way, there will be elicited a great variety of facts relating to practice, which would never be called out in any other, for the benefit of the mass. There are many who stand among our first *practitioners*, who would give oral description and explanations, that they could never be induced to write; and this is the only way in which we can ever effect a perfect interchange of views upon practical subjects, and it would constitute a most important feature of the society.

Some have been disposed to complain that the Journal was not more confined to practical matter; but it will be recollected that any detail of practice can be much more easily and perfectly given orally, than in writing. This, on the one hand, accounts for the absence, in some degree, of practical matter in the Journal, and shows the great benefit to be derived from these oral discussions of subjects. Another reason which may be noticed as also partially accounting for the comparative scarceness of practical matter in the Journal, is the fact, that, it being published by the society, and depending entirely upon voluntary contributions, its subjects would naturally be of the kind occupying the attention of, and discussed by the society; and these, as we have seen above, have been of a less practical nature than would be desirable, or than they may, and doubtless will be hereafter. Still we contend that it was, to some extent, necessary to pass over this ground to prepare the way for the future. We beg leave to notice in this place another feature of the Journal, of which some have complained. We allude to the publishing of articles (excepting bad grammar, &c.) as we receive them. The practice has been hitherto to publish all articles from contributors, deemed worthy of publication at all, without any *material* alteration. This course has been called in question, but we may remark that it is a question having *two sides.* Unless there should be a review department, it is a matter of no small difficulty to analyze every article, and present only the valuable matter. In respect to *theories*, (and this constitutes the chief source of complaint,) it would be wholly impossible, in a journal conducted on the plan of the American Journal of Dental Science, to sift and criticise every theory with which the editors might not agree. It would result in the rejection of at least half the contributions received. Not only so, but we should soon be in want of contributors, were any man, or set of men, to take upon themselves the responsibility and liberty of condemning their productions, either in whole or in part. Nor would it always be certain that the critic would be more nearly in the right than the writer. To say the least, in order to effect any respectful criticism, those undertaking to do so, would be under the necessity of making frequently a longer article than the original one. This the *editors*, situated as they are, cannot undertake. It is, however, understood by all, that the pages of the Journal are open for any discussion, conducted in a respectful and proper manner; and all are, moreover, solicited to come into the field. It might be desirable that every publication be divested of every thing not practically useful; but it has been found, in conducting most publications, that this is quite impracticable.

But suppose the editors, or a publishing committee, were to have and exercise the prerogative of using the scissors as much as they pleased, and then of declaring to the world that every article having passed this ordeal should be *read* and *believed* as it is, what would be the effect? Unless we go upon the supposition that the reviewers, as well as the review, were perfect, the effect, in our judgment, would be decidedly bad. For, in that case, all the matter coming from this source, the student is bound to believe it to be perfectly prepared to his hand, ready for his adoption and faith. Now we hold that it is the business of the dentist to keep the *teeth* in order, that they may do their *part* in the work of digestion, but to ask us to furnish the *food already digested* would be asking too much. We fear, moreover, that the stomach would perish for want of use. As the case now stands, the student does not expect to find all the matter coming to him through the Journal, freed from all incumbrance and objection; he does not expect this tree, unlike all others, to produce nuts ready cracked and freed from the shuck, but this he considers a work of his own. Even on the supposition that every theory and rule of practice could be perfect, we very much doubt whether the effect would be salutary. It would leave the mind nothing to do, save *remember*, and this would be but a poor way to cultivate the mental faculties, and prepare them for great achievements. It would be a kind of reading illy calculated to illicit talent and greatness. *False* theories are not without their good effects; they often contain facts and suggestions, which, in other hands, have led to the most important results. True, the alchemist did not find the philosopher's stone, but he did lay the foundation for modern chemistry, which has wrought greater changes in the condition of man than all other sciences combined.

Although we have occupied much more space than we intended at first, yet we can hardly forbear noticing, briefly, another topic which was agitated by the society at its last meeting; that of regulating dental practice by legislative enactment. Our own views upon this subject have been fully expressed in the first number of the fifth volume of the Journal of Dental Science, when treating upon the claims of medical science upon the dental student. Our own observation upon this subject has not led us to regard laws, at least such as have been passed hitherto, with much confidence, in the way of protecting either the profession or the public. This observation we regard too true, even as applied to the medical profession, when the chances for good results are far greater than they can be, as applied to the dental profession.

The great difficulty in making any law available, arises from the fact that it cannot be faithfully executed, for it is impossible for law to fix a standard of qualification that shall be definite. It would be difficult to pass and carry into effect any law which would *assort* those already in practice; and if not, these must be the men to carry out any law which could be passed. And if this is to be the result, it is far better there be no law. A ***licensed*** quack, of all things else, is to be despised and dreaded.

A law can go no further than to say, that all who are about to embark in the profession, shall obtain certain certificates of qualification, or shall not be authorised to practice. Now we ask who are to give these certificates? If it could be made certain that the examining committee was what it should be, the law might work good results, but we fear this, as applied to the dental profession, would be exceedingly uncertain. Suppose the law authorised state and county societies, as legally constituted examining committees, what would be the result? If it was optional, to which candidates applied, it is fair to presume that a great majority of them would seek to gain admittance in the easiest way, viz. by an examination by a county society.

Now, we venture to assert, were this to be the mode adopted in the state of New York, no course could be taken so well calculated to strengthen and perpetuate quackery.

It is beyond a doubt, that at least three-fourths of these societies would be composed of a majority of those having no claim to skill or science, save that which this law created for them; and who would, as soon as organized, nay legalized, pass resolutions declaring mineral paste to be the very best known substance for filling teeth. And if, perchance, the minority happened to disagree with them in this particular, they would be voted out as unworthy members. Such a law would create for those *now* in practice, a dignity and power that their own qualifications could never have commanded. It would enable them to propagate their exact species, and, what is far worse, enable them all to go forth under the sanction of law. It seems to us that there could be no possible way devised, by which we could so perfectly transmit into the hands of quacks, our own rights, the honor, the dignity, and the last hope of the profession. Let us beware of placing too much reliance upon law, to protect ourselves and the public. Let us rather seek to enlighten the public mind as to what constitutes good practice. Let us enable ourselves by making the American Society what it should be, to say to the public that there is *one* place from whence credentials mean what they say—one association whose certificate is a perfect guarantee for skill.

